# Neurological Manifestations and Outcomes in a Retrospective Cohort of Mexican Inpatients with SARS-CoV-2 Pneumonia: Design of a Risk Profile

**DOI:** 10.3390/healthcare9111501

**Published:** 2021-11-04

**Authors:** Silvia García, Francisco Manuel Cuatepotzo-Burgos, Christian Gabriel Toledo-Lozano, Adriana Balderrama-Soto, Sofía Lizeth Alcaraz-Estrada, Luis Montiel-López, Alberto Hilarión De la Vega-Bravo, Paul Mondragón-Terán, Maribel Santosbeña-Lagunes, Maricela Escarela-Serrano, Celia Mireya Rodríguez-Martínez, María del Carmen Méndez-Vidrio, Sandra Muñoz-López, José Alfredo Merino-Rajme, Rodrigo Alberto Rodríguez-Briseño, Fidel Cerda-Téllez, Ramón Mauricio Coral-Vázquez, Sergio Sauri-Suárez, Sandra Quiñonez-Aguilar, Juan Antonio Pineda-Juárez, Juan Antonio Suárez-Cuenca

**Affiliations:** 1 Department of Clinical Research, Centro Médico Nacional “20 de Noviembre”, ISSSTE, Mexico City 03229, Mexico; rolasil@yahoo.com.mx (S.G.); dr.francisco.manuel@outlook.com (F.M.C.-B.); drchristiantoledo@gmail.com (C.G.T.-L.); 2 Multidisciplinary Care Team for Patients with COVID-19 of Centro Médico Nacional “20 de Noviembre”, ISSSTE, Mexico City 03229, Mexico; adribalderrama@gmail.com (A.B.-S.); dr.mont79@gmail.com (L.M.-L.); alberto.delavega@issste.gob.mx (A.H.D.l.V.-B.); p.mondragonteran@gmail.com (P.M.-T.); merry6702@yahoo.com.mx (M.S.-L.); mescarela@prodigy.net.mx (M.E.-S.); mireyarod791@hotmail.com (C.M.R.-M.); mendezcmv@hotmail.com (M.d.C.M.-V.); ssanml@yahoo.com.mx (S.M.-L.); alfredo.merino@issste.gob.mx (J.A.M.-R.); rodrigo.rodriguezbr@issste.gob.mx (R.A.R.-B.); fidelct296@hotmail.com (F.C.-T.); sergiosauri@yahoo.com.mx (S.S.-S.); neuronitoq@yahoo.com.mx (S.Q.-A.); juan.pineda@issste.gob.mx (J.A.P.-J.); 3 Department of Neuroscience, Centro Médico Nacional “20 de Noviembre”, ISSSTE, Mexico City 03229, Mexico; 4 Department of Genomic Medicine, Centro Médico Nacional “20 de Noviembre”, ISSSTE, Mexico City 03229, Mexico; sofializeth@gmail.com; 5 Intensive Care Unit, Centro Médico Nacional “20 de Noviembre”, ISSSTE, Mexico City 03229, Mexico; 6 Department of Teaching and Research, Centro Médico Nacional “20 de Noviembre”, ISSSTE, Mexico City 03229, Mexico; rmcoralv@gmail.com; 7 Postgraduate Section, Escuela Superior de Medicina, Instituto Politécnico Nacional, Mexico City 11340, Mexico

**Keywords:** case fatality ratio, COVID-19, neurological symptoms, respiratory infection, risk profile

## Abstract

We analyzed the neurological manifestations in Mexican patients hospitalized with pneumonia due to COVID-19 and investigated the association between demographic, clinical, and biochemical variables and outcomes, including death. A retrospective, analytical study was conducted using the electronic records of patients hospitalized between 1 April 2020 and 30 September 2020. Records of 1040 patients were analyzed: 31.25% died and 79.42% had neurological symptoms, including headache (80.62%), anosmia (32.20%), ageusia (31.96%), myopathy (28.08%), disorientation (14.89%), encephalopathy (12.22%), neuropathy (5.4%), stroke (1.3%), seizures (1.3%), cerebral hemorrhage (1.08%), encephalitis (0.84%), central venous thrombosis (0.36%), and subarachnoid hemorrhage (0.24%). Patients also had comorbidities, such as hypertension (42.30%), diabetes mellitus (38.74%), obesity (61.34%), chronic obstructive pulmonary disease (3.17%), and asthma (2.01%). Factors associated with neurological symptoms were dyspnea, chronic obstructive pulmonary disease, advanced respiratory support, prolonged hospitalization, and worsening fibrinogen levels. Factors associated with death were older age, advanced respiratory support, amine management, chronic obstructive pulmonary disease, intensive care unit management, dyspnea, disorientation, encephalopathy, hypertension, neuropathy, diabetes, male sex, three or more neurological symptoms, and obesity grade 3. In this study we designed a profile to help predict patients at higher risk of developing neurological complications and death following COVID-19 infection.

## 1. Introduction

Coronavirus disease 2019 (COVID-19) has been defined as an acute, potentially serious respiratory infection caused by a new strain of coronavirus, the severe acute respiratory syndrome coronavirus 2 (SARS-CoV-2), a virus associated with the severe acute respiratory syndrome (SARS) family of viruses, and other viruses that cause common cold. The World Health Organization declared COVID-19 as an international health emergency on 30 January 2020, and as a global pandemic in March 2020 [1]. To date, Mexico has one of the highest disease mortality rates in the world after the United States, Brazil, and India. Thus, this pandemic is currently a great public health problem in Mexico [2].

Initially, COVID-19 was considered to eminently involve the respiratory process; however, this belief has changed since the first weeks of the pandemic as it is now known to also involve other important organs and systems, and to be very harmful for the patient [3]. Involvement of the nervous system in COVID-19 has been demonstrated [4]. It has been proposed that at least seven types of coronaviruses, including SARS-CoV-2, can affect the central nervous system (CNS) and peripheral nervous system (PNS) probably entering via olfactory nerve with relevant neurotropic mechanisms of SARS-CoV-2 [5,6].

Necropsies have confirmed brain edema and neuronal degeneration in patients who died because of COVID-19 [7]. Headache, myalgia, hypogeusia, hyposmia, dizziness, and fatigue are the most common neurological manifestations [6,8,9,10,11].

The purpose of this study was to analyze the neurological manifestations in Mexican inpatients with pneumonia caused by COVID-19 and their associations with demographic, clinical, and biochemical variables, outcomes, including death. According to the results, a profile of high-risk neurological complications and risk of death was designed.

## 2. Materials and Methods

A retrospective, longitudinal, and analytical study was conducted with COVID-19 inpatients at the Centro Nacional “20 de Noviembre” Institute for Social Security and Services for State Workers (ISSSTE) in Mexico City, from 1 April 2020, to 30 September 2020.

### 2.1. Ethical Approval

The institutional ethics committee of Centro Médico Nacional, “20 de Noviembre”, Institute for Social Security and Services for State Workers approved this study (approval no. 06-175.2020), and the study was conducted in accordance with the principles of the Declaration of Helsinki. Informed consent for participation was not required for this study in accordance with national legislation and institutional requirements.

### 2.2. Data Collection

Data were obtained from the electronic medical records of all inpatients with laboratory-confirmed SARS-CoV-2, with each record corresponding to one patient and infection. Patients who were conscious, cognitively and mentally sound, and linguistically competent provided details of disease symptoms.

The data collected included: the initial symptoms of viral disease that were onset at home, the duration of symptoms (in hours) before hospital admission, and the presenting symptoms during hospital admission.

Demographic and medical history data were also documented; electronic file, numerical identification, age, sex, occupation, address, education level, date of admission, comorbidities (hypertension, diabetes, obesity, chronic obstructive pulmonary disease [COPD], asthma), and laboratory and imaging test results were collected. Any missing data or uncertainty was clarified through direct communication with the treating clinicians.

Upon admission to the hospital, the following parameters for each patient were measured: temperature in centigrade and oxygen saturation (percentage) through a digital oximeter. Blood tests included: leukocytes per mm^2^, lymphocytes per mm^2^, platelets per mm^2^, glucose level (mg/dL), sodium level (mEq/L), creatinine level (mg/dL), blood urea nitrogen (BUN) level (mg/dL), C-reactive protein level (mg/dL), D-dimer level (micrograms per mm^2^), ferritin level (ng/mL), and fibrinogen level (mg/dL). Determinations were evaluated both at hospital admission and at the time of worst possible value during hospital stay. These variables were determined through a routine clinical laboratory auto-analyzer (Synchron CX9 PRO Clinical System; Beckman Coulter, Brea, CA, USA).

Clinical neurological manifestations assessed by a clinician including headache, encephalopathy, ageusia, anosmia, disorientation, neuropathy, myopathy, stroke, and seizures that had started at the same time as typical COVID-19 symptoms, at admission, or during hospitalization were considered as neurological symptoms.

We considered patients having severe COVID-19 as those who required advanced ventilatory support.

Documentation regarding the requirement and type of respiratory support was included for all the enrolled patients. Details regarding the requirement of intensive care unit (ICU) support and hemodialysis, and the development of acute renal failure were also documented. In patients who required advanced respiratory support, arterial blood gas levels were recorded 24 h after the procedure.

Finally, the duration of hospitalization (in days) and outcome (patient clinical condition at hospital discharge) were documented for all patients. The outcomes were classified into five categories: (1) asymptomatic; (2) respiratory symptoms; (3) only neurological symptoms; (4) respiratory and neurological symptoms, and (5) death.

### 2.3. Statistical Analyses

Descriptive analysis was performed according to the variable type. Quantitative variables with normal distribution, measures of central tendency and dispersion (mean, median, mode, range, standard deviation, and variance), and qualitative variables were reported as frequencies and percentages. Chi-square and association tests were performed (odds ratio [OR] and confidence interval [CI] 95%). A *t*-test or one-way analysis of variance (ANOVA), Bonferroni post hoc tests, and logistic regression were used as appropriate. Statistical significance was set at *p* < 0.05. Data were analyzed using SPSS Statistics 23.0 (IBM Corp., Armonk, NY, USA).

## 3. Results

### 3.1. Demographic Characteristics

A total of 1040 patients were included in this study; 64.1% were men and 35.9% were women. The mean age of all patients was 55.48 ± 14.47 years, that of male patients was 55.42 ± 14.50, and of female patients 55.59 ± 14.43 years (*p* < 0.78). A total of 832 (80%) patients were from Mexico City, 155 (14.9%) from the metropolitan area, and 53 (5.1%) from other states in Mexico; 287 (27.6%) patients were not entitled to the Centro Nacional “20 de Noviembre” ISSSTE, 164 (15.76%) were health workers, and 25 (15.24%) died.

### 3.2. Medical History and Neurological Symptoms

As shown in Table 1, neurological symptoms were observed in 79.42% patients who were further classified based on their medical history of other comorbidities. No significant differences were observed among patients for any of the comorbid conditions examined, except for COPD.

Table 2 shows the significant differences of neurological symptoms for the male/female ratio (*p* < 0.005), and the presence of only one neurological symptom (*p* < 0.003). Headache was present in 64.03% of patients with male/female ratio 399/267 (*p* < 0.001), the mean age did not affect the duration of hospitalization (*p* < 0.011), and headache was associated with the development of encephalopathy (*p* < 0.003). Anosmia was perceived in 25.57% of patients who had a difference in the length of hospitalization days (*p* < 0.005) and neuropathy development (*p* < 0.002). Ageusia was present in 264 patients who differed in the length of hospitalization days (*p* < 0.001) and neuropathy development (*p* < 0.03). Cerebral hemorrhage was identified in 9/25 (36%) patients; subarachnoid hemorrhage was present in 2/25 (8%) men, and cerebral venous thrombosis in three women (12%) (*p* < 0.04). The incidence of all types of acute cerebrovascular disease (ACD) between neurological alterations was 3.02%, (25 of 826 patients) and there was a difference in hospitalization days between patients with stroke and those without (*p* < 0.001). Disorientation was documented in 123 patients with male/female ratio 90/33 (*p* < 0.028); this symptom was associated with death (*p* < 0.0001) and prolonged hospitalization (*p* < 0.001). Encephalopathy was present in 12.22% of patients with male/female ratio 76/25 (*p* < 0.016) and prolonged hospitalization time (*p* < 0.001). Myopathy was detected in 232 patients with male/female ratio 162/70 (*p* < 0.043) and was associated with ICU management (*p* < 0.001), acute renal failure (*p* < 0.001), advanced ventilator support (*p* < 0.001), and prolonged hospitalization (*p* < 0.001). Neuropathy was present in 45 patients; of these, 46.6% died (*p* < 0.031), 16 needed ICU management (*p* < 0.001), 17 had acute renal failure (*p* < 0.001), and all these patients had prolonged hospitalization (*p* < 0.001). Seizures were observed in 11 patients and were not significantly related to COVID-19 severity. Encephalitis was documented in seven patients, three patients needed ICU management (*p* < 0.04), and they had prolonged hospitalization (*p* < 0.032).

Table 3 shows several clinical conditions and the laboratory test results on admission, as well as the worst test results during hospitalization in both groups. No evaluation was conducted for patients whose laboratory evidence was not available.

### 3.3. Outcomes

Out of all the patients, 246 (23.7%) were asymptomatic (133 men and 113 women). A total of 403 (38.8%) patients left the hospital with respiratory symptoms (253 males, 150 females), 9 (0.9%) with neurological symptoms (8 males, 1 female), 57 (5.5%) with both neurological and respiratory symptoms (44 males, 13 females), and 325 (31.3%) patients died (229 males, 96 females) (*p* < 0.001).

### 3.4. Neurological Symptoms and Outcomes

As shown in Table 4, encephalopathy, ageusia, anosmia, disorientation, neuropathy, myopathy, and stroke outcomes were statistically significant with a Chi-square test. Headache (*p* < 0.108), encephalitis (*p* < 0.269), cerebral hemorrhage (*p* < 0.138), subarachnoid hemorrhage (*p* < 0.077), central venous thrombosis (*p* < 0.987), and seizures (*p* < 0.953) were not associated with the outcomes. Furthermore, patients with neurological symptoms had longer hospitalization (13.06 ± 11.391 vs. 10.37 ± 6.71 days, *p* < 0.001).

A total of 120 patients suffered from disorientation, of whom 65 (52.84%) died (*p* < 0.001, OR 1.864 95% CI 1.532–2.268); 49 (48.51%) of 101 patients with encephalopathy died (*p* < 0.001, OR 1.651 95% CI 1.319–2.065). Of the 45 patients with documented neuropathy, 21 (46.66%) died (*p* < 0.031, OR 1.527, 95% CI 1.102–2.116). A total of 101 of 359 (28.133%) patients who had only one neurological symptom died (*p* < 0.122); 81 of 257 (31.51%) patients who had two neurological symptoms died (*p* < 0.938), and 82 of 212 (38.67%) patients with three or more neurological symptoms died (*p* < 0.010, OR 1.322, 95% CI 1.082–1.614). Of the 322 (30.96%) patients who required advanced respiratory support, 270 (83.85%) had neurological manifestations (*p* < 0.02, OR 1.084; 95% CI 1.018–1.153), and 261 (81.50%) died (*p* < 0.001, OR 9.148; 95% CI 6.933–12.069).

The clinical and demographic characteristics and case fatality ratios were analyzed. A total of 325 inpatients died (31.25%); 229 (70.46%) males and 96 (29.54%) females (*p* < 0.004, OR 1.509, 95% CI 1.138–2). A total of 159 (48.92%) of these patients were diabetic (*p* < 0.001, OR 1.514; 95% CI 1.267–1.809); 175 (53.84%) were hypertensive (*p* < 0.001, OR 1.591; 95% CI 1.329–1.905), and 24 (7.3%) had COPD (*p* < 0.001, OR, 2.433; 95% CI 1.934–3.060). Obesity was not associated with more cases of death; however, of 209 patients with obesity grade 1, 59 died (28.22%); of 273 patients with obesity grade 2, 81 died (29.67%), and of 152 patients with obesity grade 3, 60 died (39.47%) (*p* < 0.051; Table 5).

Regarding the education level and mortality of patients who died, 5 of 18 (27.77%) had no education, 75 of 183 (40.98%) had only elementary school education; 52 of 177 (29.37%) had attended secondary school; 82 of 277 (29.60%) had attended high school; 94 of 306 (30.71%) had attended college; 8 of 45 had a major (17.7%); 7 of 24 (29.16%) had a master’s degree and 2 of 10 (20%) had a doctoral degree (*p* < 0.065); 75 of 325 (23.07%) patients who died were retired.

### 3.5. Clinical Non-Neurological Conditions and Outcomes

Among 372 patients with odynophagia, 105 (28.22%) died (*p* < 0.162). Rhinorrhea was documented in 210 patients, of whom 54 (25.71%) died (*p* < 0.056, OR 1.102; 95% CI 1.005–1.209) suggesting that rhinorrhea was a protective factor against death. Dyspnea was detected in 807 patients, 285 (35.31%) of whom died (*p* < 0.001, OR 2.048; 95% CI 1.522–2.757). Of the 816 patients with fever, 265 (32.47%) died (*p* < 0.087). Of the 394 patients with chest pain, 131 (33.24%) died (*p* < 0.270). Of the 123 patients admitted to the ICU, 77 (62.60%) died (*p* < 0.001, OR 2.322; 95% CI 1.952–2.761). A total of 182 patients had acute renal failure, of whom 128 (70.32%) died (*p* < 0.001, OR 3.064; 95% CI 2.625–3.578). Finally, out of 41 patients who were on hemodialysis therapy; 32 (78.04%) died (*p* < 0.001, OR 2.661; 95% CI 2.203–3.214).

**Figure 1:**
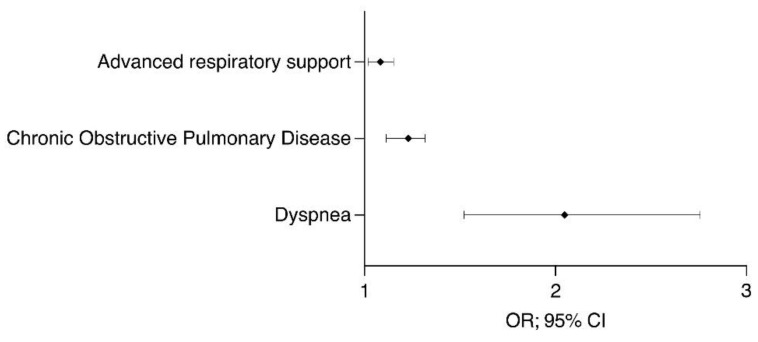
Factors associated with the presence of neurological symptoms. OR: odds ratio; CI: confidence interval.

**Figure 2:**
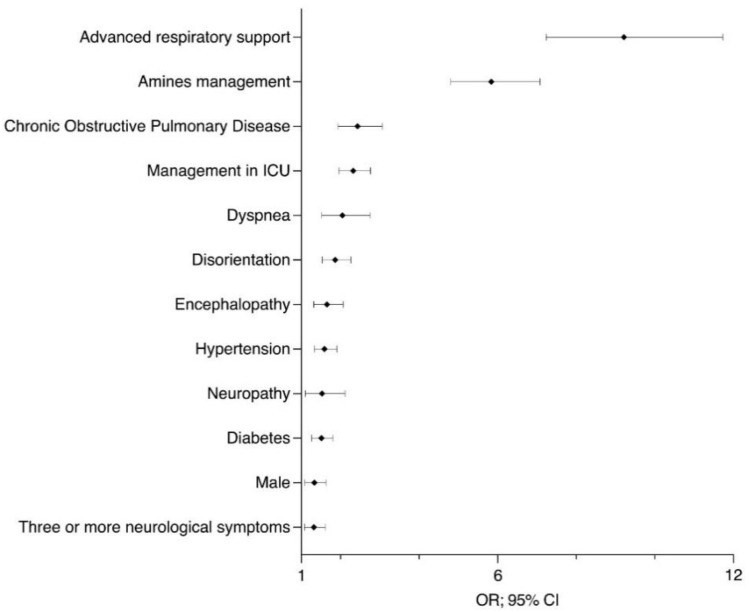
Factors associated with death. ICU: intensive care unit; OR: odds ratio; CI: confidence interval.

Based on our risk profile analysis, individuals with COPD or advanced respiratory disorders were more prone to developing neurological symptoms. The table is divided into two parts: the first includes factors associated with neurological symptoms and the second is associated with death. Each factor is arranged in descending order, depending on the OR (Table 6 and Figure 1 and Figure 2).

## 4. Discussion

Solid epidemiological data have shown that patients with COVID-19 present with frequent and diverse neurological symptoms regardless of the level of clinical severity [12,13,14], and these symptoms occurred more frequently in persons with more severe systemic presentations [15]. Currently, hospitalized patients represent a minority of COVID-19-infected cases, due to both the lack of risk factors for severe disease in many sectors of the general population and the introduction of the vaccine for SARS-CoV-2; however, non-hospitalized patients may also develop sequelae following infection, including neurological symptoms [15,16,17,18,19]. Some of these symptoms could be due to the systemic inflammatory cascade documented in these patients termed as disorders related to the nervous system, such as myopathy, neuropathy, delirium, encephalopathy, and ACD [10], and are also known as neurological complications in patients with COVID-19. There are neurological symptoms that may be related to the direct effect of SARS-CoV-2, such as hypogeusia, hyposmia, and headaches, which are called non-specific symptoms [20,21]. However, this classification is not supported by sufficient scientific evidence. In this research, all neurological manifestations contained in the electronic records were included in each case.

In this cohort, more than 1000 electronic records were analyzed. The majority of patients were men in the sixth decade of life, similar to other cohorts realized during the previous year [22,23]. More than 75% of the patients had neurological symptoms; headache was the most frequent, followed by anosmia, ageusia, myopathy, anxiety/depression, disorientation, encephalopathy, neuropathy, stroke, seizures, cerebral hemorrhage, encephalitis, central venous thrombosis, and subarachnoid hemorrhage. The percentage of neurological symptoms was lower in the first series of patients with COVID-19, possibly due to the lack of awareness to identify subtle neurological symptoms. Mao et al. [8] found neurological symptom frequency of 36.4% in 214 patients; Xiong et al. [24] reported an onset of critical neurologic symptoms in 3.5% of 917 patients, and 9.4% in those who suffered from severe or critical COVID-19. Karadaş et al. [25] found that 34.7% had neurological alterations; Kacem et al. [26] reported 72.1%, and Travi et al. [27] 30.2% with neurological symptoms.

Interestingly, in this study, neurological symptoms were documented in almost 80% of cases, which was a significantly higher incidence than that in previous studies in hospitalized Chinese [28]*,* Italian [27,28,29,30]*,* Egyptian [31], Turkish [32], Spanish [33], and American patients [34,35]. Although the absolute number of patients was mostly men, proportionally and statically, a predilection for women was observed. Headache was the most frequent neurological symptom and was prevalent among women. Remarkable subtle symptoms, such as dysgeusia and anosmia were frequent, as reported by Liguori et al. [36], who documented that 91.3% had at least one subjective neurological symptom.

The series published about neurological disturbances in COVID-19 is very diverse, heterogenous, and usually does not focus on analyses based on sex [37]; however, a few studies do [38,39,40,41]. This series suggested that men develop more severe neurological alterations than women and have a higher mortality rate, similar to this report.

Questions regarding the pathways of entry of SARS-CoV-2 in humans and its transport mode to the CNS and PNS must be answered. SARS-CoV-2 is very similar to SARS-CoV-1, both of which use spike proteins for binding to the ACE2 receptor on host cells, and transmembrane serine protease 2 is essential in this process [42]. The neuroinvasion of SARS-CoV-2 could be plausible by numerous pathways. One pathway could be the transsynaptic transfer by infected neurons derived from the olfactory nerve. This may be the cause of frequent smell alterations in patients with COVID-19. Vascular endothelium infection could explain hypertensive manifestations and leukocyte passage to the brain across the blood–brain barrier in these patients, causing central non-focal and focal neurological symptoms.

### 4.1. Non-Specific Neurological Symptoms in COVID-19

Several studies have reported that patients with COVID-19 frequently suffer from headache. Its prevalence varies according to the authors, with the time of conducting the study, and the number of patients studied. In the first studies from China [43,44,45]*,* approximately 6–13.6% of patients had a headache, while Korean authors estimated it to be 20 to 25% [46,47]. In a meta-analysis, Favas et al. [37] observed that the mean value of patients with headache was 20.2% (95% CI 19.5–20.9), very similar to that of other meta-analyses [48,49,50]. In this cohort, headache was the most frequent symptom (80.62%) and affected 71.2% of the women. Patients with headaches were found to be younger than the non-headache patients. In accordance with the global prevalence of headaches, the population of this series is likely to be more susceptible to headaches or that clinicians are more meticulous in documenting it.

Anosmia and taste disturbances are very frequent in patients with COVID-19, and their causes are still not clear [51,52]. Vaira et al. [53] reported these problems in 19.4% patients with chemosensory dysfunction. In a European multicenter study [9], the prevalence was 88% in 417 patients with mild-to-moderate disease; 12% patients had olfactory dysfunction as an initial symptom, and 18% had no nasal obstruction. In a meta-analysis, the prevalence of smell alterations was 35.8% (95% CI 21.4–50.2) and dysgeusia was 38.5% (95% CI 24.0–53.0) [43,44]; similar to the prevalence of this cohort of 32.2 and 31.96%, respectively. Both symptoms were more common in men, but without statistical significance. These symptoms were better identified by the medical team in the more recent cases. Additionally, in this cohort, 530 patients had taste disturbances or anosmia; 266 had only anosmia, 264 had dysgeusia, and 122 (23.018%) patients had both at the same time vs. 408 (76.98%) with the one or the other, thus each symptom was independent.

Initially, anosmia was attributed to ACE2 receptor disturbance in the respiratory tract along with peptide-mediated inflammation, similar to other viral airway infections. However, the inflammatory component in the airways of patients with COVID-19 is not related to the degree and duration of anosmia [9,51,54]. Damage caused by the virus to the olfactory receptor neurons is a probable explanation. Other targets proposed are the ACE2 receptor in different cells of the airway, particularly in the supporting cells, vascular pericytes of the epithelium, and olfactory bulb, which explains the long-lasting olfactory dysfunctions [55].

The causes of ageusia in COVID-19 remain unknown. The ACE2 receptor is recognized as the entry molecule for SARS-CoV-2 in human cells [56]. It is localized in the mucosa of the oral cavity and tongue [57]. ACE2 inhibitors are known to cause taste disturbances, although their mechanisms are uncertain. They do not seem to be related to alterations in blood or saliva [58,59]. It has been proposed that ACE2 inhibitors disable G-protein-coupled proteins and sodium-ion channels of the ACE2 receptor [56,60].

Similar to the Middle East respiratory syndrome coronavirus, SARS-CoV-2 can bind to the sialic acid receptor [61,62], an essential component of salivary mucin [63], that has a protective function toward the transporter glycoproteins of taste molecules within the taste pores. It is possible that SARS-CoV-2 occupies the sialic acid receptors on the taste buds, thereby increasing the degradation of taste molecules. Its reduction in saliva increases the threshold of taste. The probability of SARS-CoV-2 occupying the sialic acid receptors increases the degradation of taste molecules [63].

In accordance with the results of this cohort, most studies agree that smell alterations, taste disturbances, and headaches are the most common neurological symptoms in patients with COVID-19, but they usually do not influence the outcome or mortality. Remarkably, anosmia and ageusia were not always present together; in fact, they presented separately in more than 80% of patients, which suggests that their physiopathology mechanisms are different.

### 4.2. Neurological Complications

Neurological disturbances such as myopathy, disorientation/encephalopathy, neuropathy, stroke, seizures, cerebral hemorrhage, cerebral venous thrombosis, and subarachnoid hemorrhage are considered neurological complications of COVID-19 [14].

The incidence of all types of acute ACDs in patients with COVID-19 ranges from 0.3% to 2.1% [33,35,64,65]. In this study, of all neurological alterations, ACD was present in 3.02%, without sexual differences. This incidence was a little more frequent than the mean incidence of the other cohorts.

Stroke is the most common type of ACD in patients with COVID-19; patients with severe COVID-19 have a higher risk of stroke. Li et al. [65] conducted a study on 221 patients with COVID-19. In the study, a total of 13 (5.9%) patients presented with cerebrovascular disease, subsequently leading to COVID-19 symptoms; 84.6% of patients had stroke, one patient had cerebral venous thrombosis, and one cerebral hemorrhage. In this cohort, stroke was documented in 11 patients, who were significantly older (*p* < 0.001) and had a longer duration of hospitalization than non-stroke patients (*p* < 0.001). An interesting datum revealed that 90.9% of them were hypertensive (*p* < 0.001) in accordance with the results of other cohorts [6,35,65,66], supporting the involvement of clinical conditions in stroke’s development.

The causes of ACD in COVID-19 are not completely understood. In stroke, factors such as older age, hypertension, previous cerebrovascular events, and diabetes are closely involved in its development, similar to patients without COVID-19 [65,67]. In addition, there are specific conditions such as exacerbated inflammatory response, elevated procoagulant activity, thrombocytopenia, increased D-dimer level, and multiple organ failure [8], linked to SARS-CoV-2 or septic state, which increase the risk of ACD. Oxley et al. [68] observed that in five patients below 50 years of age who presented with stroke and COVID-19, stroke was caused due to large-vessel obstruction.

In addition, the ability of SARS-CoV-2 to bind to the ACE2 receptor on endothelial cells can cause a potential elevation in blood pressure, a condition involving both ischemic and hemorrhagic cerebral diseases, and can be aggravated by thrombocytopenia, coagulation alterations, and cytokine storms [6,69].

Viral mechanisms similar to those that occur with other viruses have also been considered, such as direct endothelial infection, subsequent vessel injury, systemic inflammatory response, thrombosis, and vasculitis [70,71]. Ding et al. [66] reported a case of severe COVID-19 and documented serious systemic vasculitis. In conclusion, the pathophysiological mechanisms involved in ACD in COVID-19 seem to be multiple and different for each patient.

In this study, encephalopathy and disorientation occurred in 21.53% of the patients, both prevalent among men and in older patients, and associated with higher mortality and prolonged hospitalization. These similarities may be both corresponding to the same pathophysiological mechanism involving clinical non-focal neurological symptoms such as delirium, disturbances of consciousness, agitation, confusion, and dysexecutive syndrome [72]. Their prevalence in patients with COVID-19 is variable, from 3.3 to 19.6% [33,73]. It has been consistent in diverse populations that older patients are more susceptible to developing it [74,75,76]; this was also confirmed in our results.

The direct invasion of CNS by SARS-CoV-2 is an encephalopathy plausible etiology. The pathway of SARS-CoV-2 arrival at the CNS could be transsynaptic by the infected cells, the olfactory nerve across the vascular endothelium, and/or leukocyte migration through the blood–brain barrier [74,77,78]. The probable incursion of CNS by SARS-CoV-2 could be implicated in the poor prognosis of encephalopathy in the acute phase of illness. Therefore, it is important to provide a detailed follow-up to patients who survive as they may develop chronic infection, which would explain the late cognitive disorders reported in COVID-19 [79,80].

In this cohort, 33.57% of the patients had myopathy and/or neuropathy, with myopathy being more prevalent. Significantly, more men were affected and none of them were associated with higher mortality. Nevertheless, they had greater severity of the disease (acute renal failure, advanced support ventilation, and prolonged hospitalization). A total of 62.5% patients who presented with myopathy and 53.33% with neuropathy left the hospital with respiratory and/or neurological symptoms that could impair the functional prognosis in this group of patients.

Neuromuscular disorders in critically ill patients have been a major problem; therefore, they have been studied extensively. Their pathophysiology is multifactorial, including metabolic disturbances, mitochondrial dysfunction, and vascular alterations that cause decrease in energy and deterioration in excitation–contraction coupling [79,81]. There is not enough evidence on the ability of SARS-CoV-2 to be neurotrophic and subsequently cause direct tissue injury [82]. It is expected that patients with severe COVID-19 will develop critical illness myopathy or polyneuropathy, similar to other etiologies [83,84].

The atrophy of the type 2 muscle fibers due to disuse causes weakness after 1 week of incapacitation and must be considered as a cause of weakness in COVID-19 [67]. Guidon and Amato [85] proposed some alternatives for the development of neuromuscular complications in COVID-19: (1) risk factors associated with SARS-CoV-2 infection cause a new neuropathy and/or myopathy (Guillain-Barré syndrome [GBS], myositis, and myopathy or polyneuropathy due to critical illness); (2) exacerbation or unmasking of previously unrecognized neuropathy (chronic inflammatory demyelinating polyneuropathy, Lambert-Eaton myasthenic syndrome, multifocal acquired demyelinating sensory and motor neuropathy, myasthenia gravis, and myositis), and degenerative disorders such as amyotrophic lateral sclerosis, spinal muscular atrophy, hereditary neuropathies, muscular dystrophies, congenital myopathies, mitochondrial myopathies, metabolic myopathies, and others) by SARS-CoV-2 infection; (3) risks associated with immunosuppressant/immunomodulation therapies, especially in patients with autoimmune neuromuscular diseases, as these patients have an increased risk and severity of COVID-19, and other infections in COVID-19 possibly caused by the immunotherapies, which might render vaccines less effective; (4) risks associated with the treatment of COVID-19 (hydroxychloroquine and chloroquine) can cause toxic neuropathy and myopathy, and also antiviral treatments (such as lopinavir/ritonavir, remdesivir), and (5) risks associated with vaccinations, such as possible inflammatory neuropathy, GBS, plexitis, and mononeuritis.

The pathophysiology of seizures in COVID-19 is complex, and its understanding could reveal new insights in this field. Diverse viruses can reach the CNS by anterograde and retrograde axonal transport [86]. In the brain, they generate reactive gliosis and trigger an inflammatory cascade activating the microglia, releasing several proinflammatory molecules, and generating neuronal hyperexcitability and apoptosis. These features play an important role in the pathogenesis of seizures. Further, there is an elevation of glutamate levels and decrease in GABA levels. Similar to another virus, SARS-CoV-2 was found in the cerebrospinal fluid of patients with COVID-19–associated viral encephalitis and possible seizures [87,88].

COVID-19 can worsen seizure control in epileptic patients and can generate seizures in patients without a history of epilepsy [89,90,91,92,93]. In a meta-analysis [37] conducted on 2043 patients with COVID-19, 23 (1.1%) had seizures (95% CI 0.7–1.7). These results were similar to the results of this cohort, where prevalence was 1.3%, with no difference between males and females, age, prolonged hospitalization, outcomes, or mortality.

Encephalitis in COVID-19 has been documented; however, its pathophysiological mechanisms are not completely understood. It has been demonstrated that SARS-CoV-2 reaches the CNS by diverse routes and is probably facilitated by a systemic hyper-inflammatory state [94,95]. A current hypothesis concerning the neuroinvasion of SARS-CoV-2 is the enteric pathway across axons of vagal afferent fibers [96]. There is sufficient evidence that encephalitis is caused by direct viral damage to the encephalon and immune-mediated neuroinflammation [97].

In this cohort, encephalitis was documented in 0.6% of the cases with neurological alterations and 0.6% of the total cases analyzed. A total of three patients (45.8%) were admitted to the ICU, and patients with encephalitis were 3.68 (1.54–8.81) times more likely to need ICU support and prolonged hospitalization.

### 4.3. Development of Neurological Non-Specific Symptoms and Complications

Headache is significantly associated with the development of encephalopathy. Cytokine storm is involved in both [98], and direct invasion of SARS-CoV-2 in the CNS has also been indicated [77,78,85]. In patients with headache and COVID-19, it is necessary to conduct close clinical surveillance to discriminate the characteristics of headache and predict the appearance of encephalopathy. Ageusia was associated with the development of disorientation, anosmia, and neuropathy. According to several researchers, taste and smell disturbances are due to effects on the peripheral nerve [37]. Hypothetically, both could be an early form of neuropathy; however, considering retrograde arrival of SARS-CoV-2 to CNS [87,99] as a plausible theory, it can be considered that afferent gustative fibers serve as a route to the CNS to explain disorientation.

### 4.4. Development of Comorbidities and Neurological Symptoms

Comorbidities in patients in this study were analogous to those of other series in different populations [46,100,101]. The most relevant findings revealed arterial hypertension in more than 40% of patients, diabetes mellitus in almost 40%, obesity in more than 60%, and COPD in a little more than 3%. Only COPD exhibited a significant association (1.23-fold increase) in the likelihood of developing neurological symptoms. Respiratory and systemic symptoms such as cough, odynophagia, rhinorrhea, dyspnea, diarrhea, and fever were associated with non-specific neurological symptoms, and all neurological and non-neurological symptoms had a common denominator: SARS-CoV-2 virus in host cells [8,20,99].

### 4.5. Outcomes

Among all patients, almost a third of them had respiratory symptoms at the time of hospital discharge, which was an important consideration for evaluation of late respiratory sequelae. Patients with arterial hypertension, diabetes mellitus, obesity, and COPD had worse outcomes [102]. Neurological manifestations, such as encephalopathy, disorientation, and neuropathy were also associated with worse outcomes.

Interestingly, no GBS cases were detected in more than 1000 patients. In the diverse cohorts, GBS was not as frequent as other neurological symptoms; this result is similar to the results of the systematic review conducted by Abu-Rumeileh et al. [103] that found only 18 patients with GBS in 14 articles that were analyzed.

The case fatality ratio was approximately 30%. Factors associated with it were: male sex, diabetes, hypertension, COPD, dyspnea, acute renal failure, hemodialysis therapy, ICU management, and requirement of advanced respiratory support. Interestingly, rhinorrhea was associated with continued existence (*p* < 0.05), which can be attributed to the fact that it is an unspecific symptom. Out of all the health workers, 15.24% died [28].

Based on the aforementioned variables, a profile of patients with a higher risk of death was designed (Table 6).

### 4.6. Limitations

Our study has several limitations. First, this was a heterogeneous population with respect to the demographic characteristics. Second, patients had different sociocultural levels. Third, all patients included were COVID-19 inpatients. Fourth, there was no continuous follow-up of the patients included in the study. Fifth, the course of non-hospitalized patients not requiring hospital care was not considered.

## 5. Conclusions

The important contributions of this study are: (1) determination of the risk for developing non-specific neurological symptoms and complications by sex was a strength of this cohort; (2) classification of the patients into five possible outcomes; (3) profile design of patients with a higher probability of developing neurological manifestations; (4) profile design of patients with a higher risk of death, and (5) possibility to differentiate a headache as an entity that increases the frequency of neurological symptoms in patients with COVID-19.

## Figures and Tables

**Table 1:** History of disease and frequency of development of neurological symptoms.

Comorbidity	Total	n (%)	Neurological Symptoms n (%)	*p* Value	OR	95% CI
Arterial hypertension	1040	440 (42.30)	388 (88.81)	0.81	0.99	0.932–1.057
Diabetes mellitus	1040	403 (38.74)	323 (80.14)	0.69	1.015	0.953–1.081
Obesity	1039	638 (61.34)	507 (79.46)	1	1.001	0.94–1.067
Grade 1	634	209 (32.96)	173 (82.77)	0.90	--	--
Grade 2	634	273 (43.05)	206 (75.45)		--	--
Grade 3	634	152 (23.97)	125 (82.83)		--	--
COPD	1040	33 (3.17)	32 (96.96)	0.007	1.23	1.149–1.317
Asthma	1040	21 (2.01)	19 (90.47)	0.2	1.142	0.991–1.317

Data are shown as frequencies, percentages. Statistical analyses were performed by Chi-square, odds ratio and 95% CI. Statistical significance: *p* < 0.05. Abbreviations: OR: odds ratio; CI: confidence interval; COPD: chronic obstructive pulmonary disease.

**Table 2:** Neurological manifestations according to sex.

Neurological Symptoms	Total	Male/Female	*p* Value	OR	95% CI
All neurological symptoms	826	512/314	0.621	0.621	0.446–0.864
One neurological symptom	359	208/151	0.003	0.666	0.512–0.867
Two neurological symptoms	257	165/92	1	1.004	0.748–1.347
Three or more neurological symptoms	210	140/72	0.522	1.113	0.810–1.529
Headache	666	399/267	0.001	0.591	0.450–0.777
Anosmia	266	172/94	0.882	1.031	0.771–1.380
Ageusia	264	182/82	0.063	1.332	0.998–1.795
Myopathy	232	162/70	0.043	1.389	1.014–1.902
Disorientation	123	90/33	0.028	1.607	1.055–2.448
Encephalopathy	101	76/25	0.016	1.790	1.118–2.867
Neuropathy	45	34/11	0.114	1.768	0.885–3.531
Stroke	11	5/6	0.216	0.462	0.140–1.524
Seizures	11	5/6	0.218	0.467	0.141–1.540
Cerebral hemorrhage	9	6/3	1	1.120	0.278–4.502
Encephalitis	7	4/3	0.706	0.744	0.166–3.343
Cerebral venous thrombosis	3	0/3	0.46	--	--
Subarachnoid hemorrhage	2	2/0	0.540	--	--

Data are shown as frequencies, percentages. Statistical analyses were performed by Chi-square, Odds ratio and 95%CI. Statistical significance: *p* < 0.05. Abbreviations: OR, Odds Ratio; CI, Confidence Interval.

**Table 3:** Several clinical conditions and results of laboratory tests at admission and their worst levels during hospitalization.

	With Neurological Symptoms (n = 826)	Without Neurological Symptoms (n = 214)	*p* Value
O_2_ saturation at admission (%)	83.3 ± 13.17	84.51 ± 11.73	0.122
Symptoms duration before admission (hours)	179.08 ± 125.112	162.07 ± 114.597	0.381
Hospital stay (days)	13.06 ± 11.391	10.37 ± 6.718	0.001
Data at admission Leukocytes (cells per mm^2^) Lymphocytes (cells per mm^2^) Platelets (cells per mm^2^) Plasma glucose (mg/dL) Plasma sodium (mEq/L) Plasma creatinine (mg/dL) BUN (mg/dL) Plasma C-reactive protein (mg/dL) Plasma D-dimer (μg/mL) Plasma ferritin (ng/mL) Plasma fibrinogen (mg/dL)	9109.547 ± 4795.254 1202.938 ± 1149.151 233369.829 ± 100659.543 135.724 ± 75.294 138.08 ± 4.778 1.213 ± 1.541 23.799 ± 20.041 113.039 ± 95.982 4.584 ± 22.327 1078.132 ± 1219.426 510.623 ± 169.637	9053.545 ± 4244.95 1098.591 ± 614.295 250575 ± 98015.044 130.121 ± 66.693 137.234 ± 10.202 1.13 ± 1.392 22.402 ± 19.407 118.202 ± 101.831 2.699 ± 5.447 1209.628 ± 1780.672 550.259 ± 376.028	0.091 0.144 0.497 0.165 0.138 0.254 0.256 0.408 0.074 0.215 0.508
Wost data during hospitalization Plasma creatinine (mg/dL) BUN (mg/dL) Plasma C-reactive protein (mg/dL) Plasma D-dimer (μg/mL) Plasma ferritin (ng/mL) Plasma fibrinogen (mg/dL)	1.488 ± 1.973 30.298 ± 31.362 99.389 ± 99.746 6.392 ± 25.452 1548.107 ± 3796.142 493.983 ± 170.579	1.374 ± 1.555 30.949 ± 37.025 97.915 ± 102.012 8.889 ± 32.111 1241.529 ± 1159.54 505.096 ± 117.984	0.105 0.410 0.766 0.130 0.408 0.026

Data are shown as mean and standard deviation. Statistical analyses were performed by one-way, independent, *t*-test. Statistical significance: *p* < 0.05. Abbreviations: BUN: blood urea nitrogen.

**Table 4:** Neurological symptoms during hospitalization and clinical outcomes at hospital discharge.

	Type of Clinical Outcome at Hospital Discharge					
	Asymptomatic	Only Respiratory Symptoms	Only Neurological Symptoms	Neurological and Respiratory Symptoms	Death	*p* Value
Neurological Symptoms during hospitalization Headache	166	242	5	43	210	0.108
Encephalopathy	9	16	3	24	49	<0.001
Ageusia	53	92	4	29	86	<0.001
Anosmia	61	92	4	27	82	0.001
Disorientation	11	18	3	26	65	<0.001
Neuropathy	0	5	2	17	21	<0.001
Myopathy	7	104	5	36	80	<0.001
Stroke	1	4	1	1	4	0.039
Seizures	2	5	0	1	3	0.953

Data are shown as frequencies. Statistical analyses were performed by Chi-square. Statistical significance: *p* < 0.05.

**Table 5:** Outcomes according to previous comorbidities.

Comorbidity	N (%)	1	2	3	4	5	*p* Value
Arterial hypertension	440 (42.30)	73	164	3	25	175	<0.001
Diabetes mellitus	403 (38.74)	66	149	1	28	159	<0.001
Obesity	638 (61.34)	131	273	2	33	200	0.001
Grade 1	209 (32.96)	58	78	1	13	59	0.026
Grade 2	273 (43.05)	48	130	1	13	81	
Grade 3	152 (23.97)	23	63	0	6	60	
Chronic obstructive pulmonary disease	33 (3.17)	3	4	0	2	24	<0.001
Asthma	21 (2.01)	7	7	1	1	5	0.270

Data are shown as frequencies and percentages. Statistical analyses were performed by Chi-square test. Statistical significance: *p* < 0.05. Codes: 1: asymptomatic; 2: respiratory symptoms; 3: neurological symptoms; 4: neurological and respiratory symptoms; 5: death.

**Table 6:** Profile of factors associated with neurological symptoms and death.

Factors Associated with Neurological Symptoms	*p* Value	Analysis
Dyspnea	0.0001	OR; 95% CI 2.048; (1.522–2.7579)
Chronic obstructive pulmonary disease	0.0070	1.23; (1.114–1.317)
Advanced respiratory support	0.0200	1.084; (1.018–1.153)
	Mean Difference	
* Prolonged hospitalization	0.0001	13.18 ± 12.69 vs. 10.37 ± 6.72
* Worst fibrinogen level (mg/dL)	0.0260	493.981 ± 70.58 vs. 505.10 ± 17.98
Factors associated with death		
		OR; 95% CI
Advanced respiratory support	0.0001	9.212; (7.234–11.730)
Amine management	0.0001	5.835; (4.804–7.087)
Chronic obstructive pulmonary disease	0.0001	2.433; (1.934–3.060)
Management in intensive care unit	0.0001	2.322; (1.952–2.761)
Dyspnea	0.0001	2.048; (1.522–2.757)
Disorientation	0.0001	1.864; (1.532–2.268)
Encephalopathy	0.0001	1.651; (1.319–2.065)
Hypertension	0.0001	1.591; (1.329–1.905)
Neuropathy	0.0300	1.527; (1.102–2.116)
Diabetes	0.0001	1.514; (1.267–1.809)
Male sex	0.0040	1.334; (1.090- 1.632)
Three or more neurological symptoms	0.0100	1.322; (1.082–1.614)
Obesity grade 3	0.0231	1.533; (1.074–2.188)
		Mean Difference
* Older age	0.0001	61.63 ± 12.57 vs. 52.68 ± 14.42

* Numeric variables. Data are shown as mean, standard deviation, odds ratio, and confidence interval. Statistical analyses were performed by one-way, independent, *t*-test, and logistic regression about factors related to neurological symptoms and death. Statistical significance: *p* < 0.05. Abbreviations: OR, odds ratio; CI, confidence interval.

## Data Availability

Datasets analyzed or generated during the study can be requested from the authors.
